# Group consensus peer review in radiation oncology: commitment to quality

**DOI:** 10.1186/s13014-018-1006-1

**Published:** 2018-03-27

**Authors:** W Neil Duggar, Rahul Bhandari, Chunli Claus Yang, Srinivasan Vijayakumar

**Affiliations:** Radiation Oncology Department, University of Mississippi Medical Center, 350 W. Woodrow Wilson, Suite 1600, Jackson, MS 39213 USA

## Abstract

**Background:**

Peer review, especially prospective peer review, has been supported by professional organizations as an important element in optimal Radiation Oncology practice based on its demonstration of efficacy at detecting and preventing errors prior to patient treatment. Implementation of peer review is not without barriers, but solutions do exist to mitigate or eliminate some of those barriers.

**Methods:**

Peer review practice at our institution involves three key elements: new patient conference, treatment planning conference, and chart rounds. The treatment planning conference is an adaptation of the group consensus peer review model from radiology which utilizes a group of peers reviewing each treatment plan prior to implementation. The peer group in radiation oncology includes Radiation Oncologists, Physician Residents, Medical Physicists, Dosimetrists, and Therapists. Thus, technical and clinical aspects of each plan are evaluated simultaneously.

**Results:**

Though peer review is held in high regard in Radiation Oncology, many barriers commonly exist preventing optimal implementation such as time intensiveness, repetition, and distraction from clinic time with patients. Through the use of automated review tools and commitment by individuals and administration in regards to staffing, scheduling, and responsibilities, these barriers have been mitigated to implement this Group Consensus Peer Review model into a Radiation Oncology Clinic.

**Conclusion:**

A Group Consensus Peer Review model has been implemented with strategies to address common barriers to effective and efficient peer review.

## Background

Peer review, and specifically prospective in nature, has been demonstrated as an important quality mechanism in the field of Radiation Oncology and is championed by various professional organizations such as ACR and ASTRO based on consistent efficacy at prevention of minor and major errors [1–4]. Just as assuring exceptional quality of care is important to any medical specialty, it is also of great importance to Radiation Oncologists from the time of consult to subsequent follow-up appointments. Without quality assurance and control, treatment-related errors have the potential for causing serious injury to the patient or subpar treatment results that could have been avoided with appropriate systems in place.

In the realm of radiation treatment planning, a major potential cause of error is inter-provider variation. One of the greater risks of treatment-related errors pertains to the more subjective decisions that radiation oncology physicians have to make, such as deciding on target volumes, target margins, normal tissue contours, critical organ dose limits, beam arrangements, prescription doses, and treatment delivery techniques. In light of this, the room for error quickly widens if an adequate peer review process is not established. Furthermore, the inherent complexities of different sites of disease (i.e. when evaluating head and neck plans versus breast plans) can also contribute to the adaptations necessary for the peer review process [5–9].

Facilitating treatment plans with a peer review process has proven to be beneficial throughout several studies. Boxer, et al. [10] showed that real-time peer reviewing audit on patient management resulted in approximately 6% failed plans that did not pass the audit process. In the same way, in a large Ontario study, Brundage, et al. [6] revealed that around 7% of the cases were found to have errors through a real-time peer review audit of 1000 patients. In a more recent systematic review of published data pertaining to peer-reviews of radiation therapy plans, Brunskill, et al. [11] concluded that there were plan modifications in about 10.8% of the cases. Major changes were found in approximately 1.8% of the cases, with the majority of the recommended changes concerning target volume delineations. Consistent with the findings mentioned above, Hoopes, et al. [3] reported that when 600 oncologists were questioned on the effect of the peer review process in their own practices, 90% of the study respondents claimed that modifications had to be made in 7–10% of the treatment plans.

Barriers to effective peer review exist such as repetition, time intensiveness, and distraction from clinic time with patients [12]. These barriers must be overcome by strategies increase not only the effectiveness, but also the efficiency of peer review. An effective and efficient peer review process is executed with the help of a well-structured quality assurance program spanning from computed tomography (CT)-simulation to treatment delivery, which minimizes variations and standardizes procedures. Increasing automated tools and processes can assist in overcoming barriers related to time in busy clinics [13–15].

Due to increasing complexity in Radiation Oncology and its treatment techniques, synchronous evolution of quality review is also of great importance. In respect to our institution’s peer review process, this process is continually evolving. A group consensus peer review model, which has recently proved successful in radiology at Massachusetts General, has been implemented, but also requires significant commitment by the participating departments and the individual team members [16].

## Methods

### Peer review practice

In respect to our Institution’s peer review process, we use specific strategies in hopes to decrease radiation treatment planning-related errors. Peer review of treatment plans consists of a three-step process before a stamp of approval is given to a specific plan testifying to its efficacy and safety. The review process is performed in the context of a multidisciplinary team that includes physicians, residents, physicists, and dosimetrists. This mode of peer review process ensures our department is providing consistent standards of practice, along with allowing the parties involved in planning to better prioritize and optimize their workload.

The first stage of peer review occurs for patients at “New Patients Case Conference” during which time a discussion is held about each new patient seen in the Department the follow. This conference occurs once a week at 8 am when the clinical schedule has been protected. During the conference, each patient’s history is reviewed briefly in addition to treatment intentions. The goals of the conference include recognition of proper diagnosis procedures, remaining work-up, appropriate treatment intent, development of comprehensive treatment plan, and finally, whether any radiation therapy treatment has justified intention, prescription and technique.

The second peer review stage is unique to this institution and has been described in the field of radiology [16] as “Peer Review by Group Consensus.” Any case that is considered complex (i.e. IMRT, VMAT, SBRT, pediatric, retreatment, rare disease entity, etc.) must go through a multi-disciplinary treatment planning conference, which includes physicians, residents, physicists, and dosimetrists, to collectively analyze treatment plans via presentation software/hardware and remote conferencing with another department site. At least two physicians besides the patient’s attending physician are required for the case to be considered reviewed. Additionally, all available physician residents and the on-call physicist attend the conference. The planners, or their designees, are present during presentation of their respective treatment plans. Approximately 70% of our cases go through this planning conference. The use of multi-disciplinary peer review allows the physical and practical portions of the treatment plans to be evaluated in addition to the clinical components. During the peer review of a treatment plan, the gross tumor volume (GTV), clinical target volume (CTV), and planning target volumes (PTV) are first evaluated and either agreed or disagreed upon, while the relevant clinical information is shared by the attending and/or covering physician resident. Next, automated DVH analysis is reviewed based on color-coding and numerical results. With this automated information, the physicians can now determine whether further investigation of the plan needs to be pursued depending on what constraint is not passing. Finally, the isodose lines are evaluated in relation to coverage, homogeneity, proximity to organs at risk (OAR), and relationship to unspecified normal tissue regions. Plans are either collectively approved or sent back for re-planning. Any disagreement goes to a vote mediated by the chairperson of the department.

The third and final stage of peer review is the use of weekly chart rounds to evaluate all patients who have started a new treatment plan including new starts, boosts, brachytherapy, plan modifications, etc. During these rounds, documentation such as consent and pathology are reviewed in addition to portal imaging and cone beam CT shift trends. The overall appropriateness of the dose, treatment technique, and patient tolerance to treatment are also discussed. Any major unplanned interruptions or attendance issues are collectively reviewed for solutions and a morbidity and mortality review occurs after chart rounds for any patients who passed during the last week for identification of any potential role of radiation oncology or any other learning opportunity.

## Results

The “Peer Review by Group Consensus” model is unique in the Radiation Oncology clinic, and several logistical challenges, such as scheduling, communication, and workflow, had to be overcome in order to implement this model of peer review. Two conferences are scheduled each week at 8:30 am on Tuesdays and Thursdays and the clinician schedules are blocked for one hour. This allows a rhythm for planners and physicians to prepare their plans in time for these conferences. Once the attending is satisfied with the treatment plan, it is designated for the next conference. If a plan needs peer review and an unacceptable delay will result from waiting on these conferences, then an ad hoc conference may be added by the attending physician either during a lunch hour or at the end of chart rounds which is also protected time. The two campuses of the department are connected by video conference and the treatment plans are shown simultaneously to both campuses utilizing the desktop sharing feature of Microsoft Lync. Though the goal of the model is to have all patients to be reviewed prior to first treatment, exceptions have been made to this to prevent unacceptable delays to patient start time. Most patients are reviewed prior to first fraction and all patients are reviewed before they reach three treatment fractions. The exception to this rule would be stereotactic or hypofractionated patients which are required to be reviewed before the first fraction. Once peer review is completed, the physics staff will perform the more common, but also more detailed pre-treatment check and then place the patient on the therapists list for scheduling.

Numerical statistics are not currently available for the plans which are rejected by the conference for re-plan, but once a plan is reviewed it conference, it receives one of three designations: approved, approved with changes, or re-plan with changes. If one of the first two are achieved, then the plan does not have to go back through conference, but if the result was rejection for re-planning, then the case must be presented again. The most common modifications recommended when designated as “approved with changes” by the conference are fractionation schedule (i.e. 180 cGy instead of 200 cGy per day). When plans require re-planning and representation, usually a change to the treatment volumes was required, but has also been recognition of inadequate attention to special concerns such as beam energy with either pediatrics or pacemakers.

## Discussion

Quality assurance performs at its optimum level with high levels of commitment, appropriate staffing, and enough allotted time to adequately perform peer review of each case of interest. Caissie, et al. [17] showed that only 52.3% of the programs surveyed performed a peer review of > 80% of curative treatment plans due to barriers to peer review included time constraints (27.3%) and radiation oncologist availability (34.1%). This study demonstrates that successful implementation of a peer review system requires commitment not only from clinical staff, but also those making administrative decisions about scheduling, responsibilities, and staffing.

These barriers are overcome by demonstrating commitment to the Group Consensus model with creative scheduling of designated times where this group consensus peer review takes place. Additionally, ad hoc sessions may be called at times where time is already blocked as well, such as after chart rounds. Efforts to enhance efficiency in plan review can also increase the feasibility of implementing a similar model. Here, automated DVH analysis is utilized with a quick color-coded pass/fail system for department constraints which greatly accelerates review of the DVH for adherence to department criteria.

## Conclusion

Peer review in a Radiation Oncology is not only a noble endeavor, but is essential. Due to the challenging nature of its implementation, efficiency and efficacy must be essential ingredients to any peer review models. This institution considers all members of the radiation therapy treatment planning team as essential to the peer review process leading to inclusion in the “Group Consensus Peer Review” model within treatment planning conferences which are made feasible through committed and creative scheduling and automated methods of plan presentation. This Group Consensus model has demonstrated efficacy in other disciplines and represents a novel form of peer review in Radiation Oncology clinics [16].

## Abbreviations

ACR: American College of Radiology
ASTRO: American Society for Radiation Oncology
CT: Computed Tomography
CTV: Clinical Target Volume
DVH: Dose Volume Histogram
GTV: Gross Tumor Volume
IMRT: Intensity Modulated Radiation Therapy
OAR: Organ at Risk
PTV: Planning Target Volume
SBRT: Stereotactic Body Radiation Therapy
VMAT: Volumetric Modulated Arc Therapy
